# Comprehensive Assessment of the STIMs and Orais Expression in Polycystic Ovary Syndrome

**DOI:** 10.3389/fendo.2022.874987

**Published:** 2022-05-20

**Authors:** Tian Song, Ping Li, Qiumin Wang, Baozhen Hao, Ying Wang, Yuehong Bian, Yuhua Shi

**Affiliations:** ^1^ Center for Reproductive Medicine, Cheeloo College of Medicine, Shandong University, Jinan, China; ^2^ Key laboratory of Reproductive Endocrinology of Ministry of Education, Shandong University, Jinan, China; ^3^ Shandong Key Laboratory of Reproductive Medicine, Jinan, China; ^4^ Shandong Provincial Clinical Research Center for Reproductive Health, Jinan, China; ^5^ National Research Center for Assisted Reproductive Technology and Reproductive Genetics, Shandong University, Jinan, China; ^6^ Department of Reproductive Medicine, Women and Children’s Hospital, School of Medicine, Xiamen University, Xiamen, China; ^7^ Xiamen Key Laboratory of Reproduction and Genetics, Xiamen, China; ^8^ Shandong Provincial Maternal and Child Health Care Hospital, Cheeloo College of Medicine, Shandong University, Jinan, China; ^9^ Guangdong Provincial People’s Hospital, Guangzhou, China

**Keywords:** polycystic ovary syndrome, store-operated Ca2+ entry, STIM1, Orai1, STIM2

## Abstract

**Background:**

Polycystic ovary syndrome (PCOS) is a heterogeneous endocrine disease characterized by irregular menstrual, hyperandrogenism, and polycystic ovaries. The definitive mechanism of the disorder is not fully elucidated. Store-operated Ca2+ entry (SOCE) plays a role in glucose and lipid metabolism, inflammation, hormone secretion, and cell proliferation. STIMs and Orais are the main elements of SOCE. The potential role of SOCE in PCOS pathogenesis remains unclear.

**Methods:**

The expression of STIMs and Orais in granulosa cells (GCs) derived from 83 patients with PCOS and 83 controls were analyzed, respectively, by using quantitative reverse transcription polymerase chain reaction. Binary regression analysis was used to identify the factors affecting PCOS after adjusted by body mass index and age. Pearson correlation analysis was used to determine the association between PCOS phenotypes and SOCE genes expression.

**Results:**

Significantly increased expression of STIM1, STIM2, Orai1, and Orai2 were observed in patients with PCOS compared with controls (*P* = 0.037, *P* = 0.004, *P* ≤ 0.001, and *P* = 0.013, respectively), whereas the expression of Orai3 was decreased (*P* = 0.003). In addition, the expression levels of STIMs and Orais were identified as the factors affecting PCOS (*P* < 0.05). The expressions of these genes were correlated with hormone level and antral follicle count (*P* < 0.05).

**Conclusions:**

For the first time, our findings indicated that the elements of SOCE were differently expressed, where STIM1, STIM2, Orai1, and Orai2 significantly increased, whereas Orai3 decreased in PCOS GCs, which might be dominantly involved in dysfunction of ovarian GCs and hormonal changes in PCOS.

## Introduction

Polycystic ovary syndrome (PCOS) is a heterogeneous endocrine disease that affects approximately 4%–21% of reproductive age women worldwide (1). It could be diagnosed by irregular menstrual, hyperandrogenism, and polycystic ovarian morphology (1, 2). In addition to female infertility, a wide range of pathological symptoms, such as insulin resistance, obesity, diabetes mellitus type II (T2DM), and hypertension, are common in women with PCOS (2, 3). Literature suggests that genetic and environmental factors contribute to the syndrome in a complicated manner (4, 5). However, the definitive mechanism is not fully elucidated.

Calcium (Ca2+) takes essential part in various cellular functions, signaling pathways, and metabolic processes in all tissues as a second messenger (6). Maintaining calcium homeostasis is crucial in physiological activities, whereas dysregulation of Ca2+ signals has been associated with some of the major diseases in humans such as cardiac disease, Alzheimer’s disease, pancreatitis, and asthma (7–10).

Store-operated Ca2+ entry (SOCE) is a fundamentally important mechanism for maintaining calcium homeostasis, which composes of endoplasmic reticulum (ER) Ca2+-binding protein stromal interaction molecules (STIMs) and Ca2+ release–activated Ca2+ channel proteins (ORAIs). STIM1 and Orai1 are the main elements of SOCE. Once the ER Ca2+ store depletion, STIM1 is activated to couple to open Orai1 channel to refill ER-luminal Ca2+ storage and then regulate the subsequent cellular physiological processes (11–14). In addition, recent studies have reported that dysfunctional SOCE in β-cell contributes to diabetes pathogenesis (15) and defined SOCE as a critical Ca^2+^ signaling pathway that controls lipid metabolism in mouse and human cells (16), as well as in immunity (17) and inflammation (18) processes. Women with PCOS have an increased risk of chronic diseases such as T2DM and obesity (2). It is reasonable to hypothesize whether abnormal SOCE will have a long-term effect on PCOS. Moreover, dysfunction of ovarian granulosa cells (GCs) was estimated as an important element of the arrest of follicle growth, which was demonstrated to be the underlying mechanism of etiology of PCOS (19, 20). Few studies have been conducted to determine the association between SOCE and PCOS, and it is assumed that the main elements of SOCE show differential expression in PCOS, which may then transmit aberrant regulatory signaling in cellular processes and result in the functional abnormality of GCs. Therefore, in this study, we aimed to investigate the trends of core factors of the SOCE signaling pathway differentially expressed in GCs between the patients with PCOS and controls to partly explain the pathophysiology of the disease.

## Material and Methods

### Patients and Granulosa Cell Collection

We collected GCs from follicular fluid of 166 included participants (83 patients with PCOS and 83 controls) undergoing oocyte retrieval for in vitro fertilization (IVF) or intracytoplasmic sperm injection (ICSI) between 2019 and 2021 at the Center for Reproductive Medicine, Shandong University. Women with PCOS were diagnosed according to the revised Rotterdam consensus (21). Any two of the following three criteria were required: (1) oligo- and/or anovulation; (2) clinical and/or biochemical signs of hyperandrogenism; and (3) polycystic ovaries, etiologies such as Cushing syndrome, delayed adrenocortical hyperplasia, and adrenal androgen-secreting tumors were excluded. Women with regular menstruation, no endocrine abnormalities and normal ovarian morphology were included as controls. The patients were all aged 20 to 40 years, and we controlled their body mass index (BMI) under 30 kg/m^2^. Moreover, we excluded patients with ovarian surgery history, reproductive system abnormalities, chromosomal diseases, and metabolic disorders that might affect pregnancy outcomes. This study was approved by the institutional review board of the Center for Reproductive Medicine, Cheeloo College of Medicine, Shandong University with approval number 132. The clinical and endocrine parameters of PCOS and control groups were analyzed. GCs from follicular fluid of patients were collected as described previously (22) and then immediately stored at −80°C for further analysis.

### Clinical Phenotype Measurement and Ultrasonography

Endocrine hormones including anti-Mullerian hormone (AMH), follicle-stimulating hormone (FSH), luteinizing hormone (LH), estradiol (E2), and total testosterone (T) detected using venous blood on days 2–4 of the menstrual cycle were retrospectively analyzed. Moreover, after 12 h of overnight fasting, venous blood was collected to measure fasting insulin (FINS) and plasma glucose (GLU). The homeostasis model assessment of insulin resistance (HOMA-IR) was calculated by the following equation: HOMA-IR = GLU (mmol/L) × FINS (mIU/L)/22.5. Transvaginal ultrasonography was routinely conducted. The antral follicle count (AFC) was defined as the number of bilateral follicles (2–10 mm in diameter) in the early follicular phase.

### Total RNA Extraction and Real-Time PCR

To extract total RNA from GCs, TRIzol Reagent (Life Technologies, Shanghai) was used following the manufacturer’s instructions. Briefly, chloroform was added to TRIzol (1:5) for lysis, then incubated at room temperature for 2–3 min, and centrifuged at 12,000 × g at 4°C for 15 min, and the upper aqueous phase containing the RNA was processed by precipitation and elution to obtain pure RNA. Analyzing the quality of RNA by a NanoDrop 2000 (Thermo Fisher Scientific, USA) at the absorbance of 260 nm/280 nm. According to the manufacturer’s protocol, RNA (1 μg) was used to synthesize the corresponding cDNA using the Prime Script RT Reagent Kit with gDNA Eraser (TaKaRa, China). Then, cDNA amplification was performed by reverse transcription polymerase chain reaction (RT-PCR) in replicate using Quanti Nova SYBR Green PCR Kit (QIAGEN, Germany) following the manufacturer’s instructions. Because the amount of RNA that we obtained were not enough to verify in multiple target genes at the same time, we randomly enrolled 100 participants in each group as much as possible for subsequent RT-PCR experiment of the five genes, and the verification cohort was 50 versus 50. The PCR primers for RT-PCR were all listed in **Table 1**. mRNA levels were normalized against the corresponding levels of glyceraldehyde-3-phosphate Dehydrogenase (GAPDH) mRNA, which served as a housekeeping gene. The relative expression of genes was calculated using the 2^–ΔΔCT^ method and expressed as a fold change relative to that of the controls.

**Table 1:** Sequences of primers for RT-PCR.

Gene	Primer Sequences
STIM1	F : AGTCACAGTGAGAAGGCGAC
	R: CAATTCGGCAAAACTCTGCTG
Orai1	F : GGACGCTGACCACGACTAC
	R : GGGACTCCTTGACCGAGTT
STIM2	F : GGAGAGGCTTGAAAAGGCAC
	R : CCTTAGCTCTCTCAGCCGAC
Orai2	F : CATCCACTTCTACCGCTCCC
	R : CAGCTGGACCTTGAGCTTGT
Orai3	F : CCTGGTTGGTTGGGTCAAGT
	R : CAGAGGACCGTGGGAGATTG
GAPDH	F: GCACCGTCAAGGCTGAGAAC
	R: TGGTGAAGACGCCAGTGGA

STIM1, stromal interaction molecule 1; STIM2, stromal interaction molecule 2; Orai1, calcium release-activated calcium modulator1; Orai2, calcium release-activated calcium modulator 2; Orai3, calcium release-activated calcium modulator 3.

### Statistical Analysis

Statistical analysis was performed using SPSS 21.0 (SPSS, Chicago, IL, USA). Kolmogorov–Smirnov test was used to test for normality of continuous variables. The student’s *t*-test was used to analyze normally distributed variables to determine statistical significance, and Mann-Whitney *U*-test was used to assess non-parametric data. Binary logistic regression analysis was use to assess the risk factors of PCOS. Correlation between different variables was analyzed by Pearson correlation analysis. Value of *P* < 0.05 was considered statistically significant.

## Results

### Clinical Characteristics of Patiens with PCOS and Controls

The clinical characteristics of the 83 patients with PCOS and 83 controls were shown in **Table 2**. Data were presented as mean ± SD. Compared to controls, BMI (*P* < 0.001), AMH (*P* < 0.001), LH (*P* < 0.001), T (*P* < 0.001), and AFC (*P* < 0.001) were significantly increased in PCOS group while FSH (P=0.003) was significantly decreased. In addition, the corresponding clinical baseline characteristics of each gene group (50 patients with PCOS versus 50 controls) were shown in **Supplementary Table 1**.

**Table 2:** Clinical and endocrine parameters of patients with PCOS and controls.

Basic parameters	PCOS (n = 83)	Control (n = 83)	*P*-value
Age (years)	30.1 ± 4	30.56 ± 4.35	NS
BMI (kg/m^2^)	24.84 ± 3.44	22.84 ± 3.45	<0.001
AMH (ng/ml)	8.08 ± 4.11	3.99 ± 1.85	<0.001
LH (IU/L)	8.92 ± 4.32	6.18 ± 7.19	<0.001
FSH (U/L)	5.71 ± 1.60	6.18 ± 1.61	0.003
T (ng/dL)	36.27 ± 13.48	25.05 ± 11.25	<0.001
E2 (pg/mL)	39.68 ± 18.68	40.02 ± 18.08	NS
GLU (mmol/L)	5.31 ± 0.40	5.24 ± 0.38	NS
Fasting Insulin (mIU/L)	16.37 ± 7.35	15.6 ± 13.56	NS
HOMA-IR	3.92 ± 1.82	3.63 ± 3.24	NS
AFC	24.76 ± 7.92	16.15 ± 5.85	<0.001

Data were presented as mean ± SD. BMI, body mass index; AMH, antimullerian hormone; LH, luteinizing hormone; FSH, follicle-stimulating hormone; E2, estradiol; T, testosterone; GLU, glucose; AFC, antral follicle count. NS, not statistically significant.

### The Expression of STIMs and Orais in PCOS GCs

**Figure 1:**
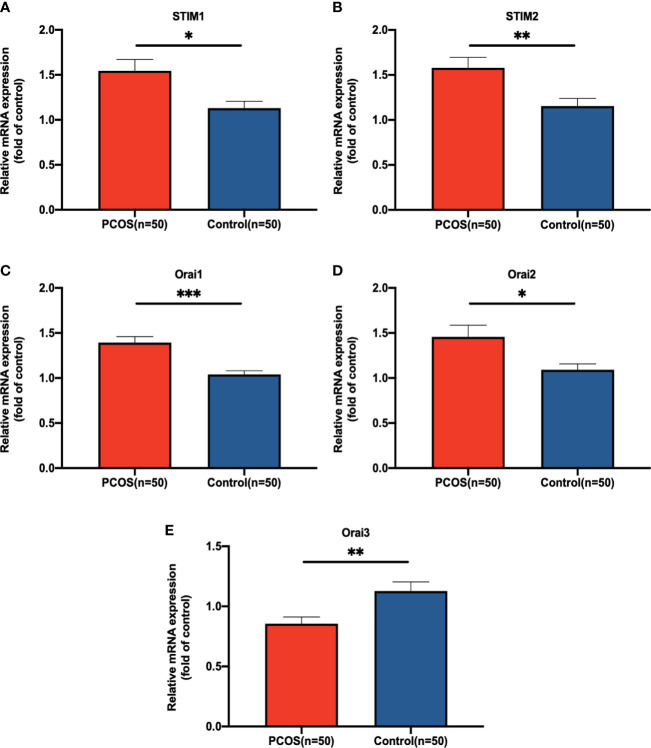
The relative expression level of **(A)** for STIM1, **(B)** for STIM2, **(C)** for Orai1, **(D)** for Orai2, and **(E)** for Orai3 in GCs of patients with PCOS. The expression level was detected by qRT-PCR and normalized against GAPDH. Statistical analysis was performed by Student’s t-test. Data were shown as the mean ± SEM (standard error of mean). *P < 0.05, **P < 0.01, and ***P ≤ 0.001.

To verify the activation of SOCE signaling, expression levels of core components in the SOCE signaling pathway were examined by RT-PCR between 50 patients with PCOS and 50 control patients, respectively. Using GAPDH as a housekeeping gene. As shown in **Figure 1**, the expression of STIM1, STIM2, Orai1, and Orai2 were significantly increased in PCOS group (*P* = 0.037, *P* = 0.004, *P* ≤ 0.001, and *P* = 0.013, respectively), and Orai3 was significantly decreased in GCs of patients with PCOS (*P*=0.003).

### Binary Logistic Regression Analysis of Gene Expression in PCOS

**Figure 2:**
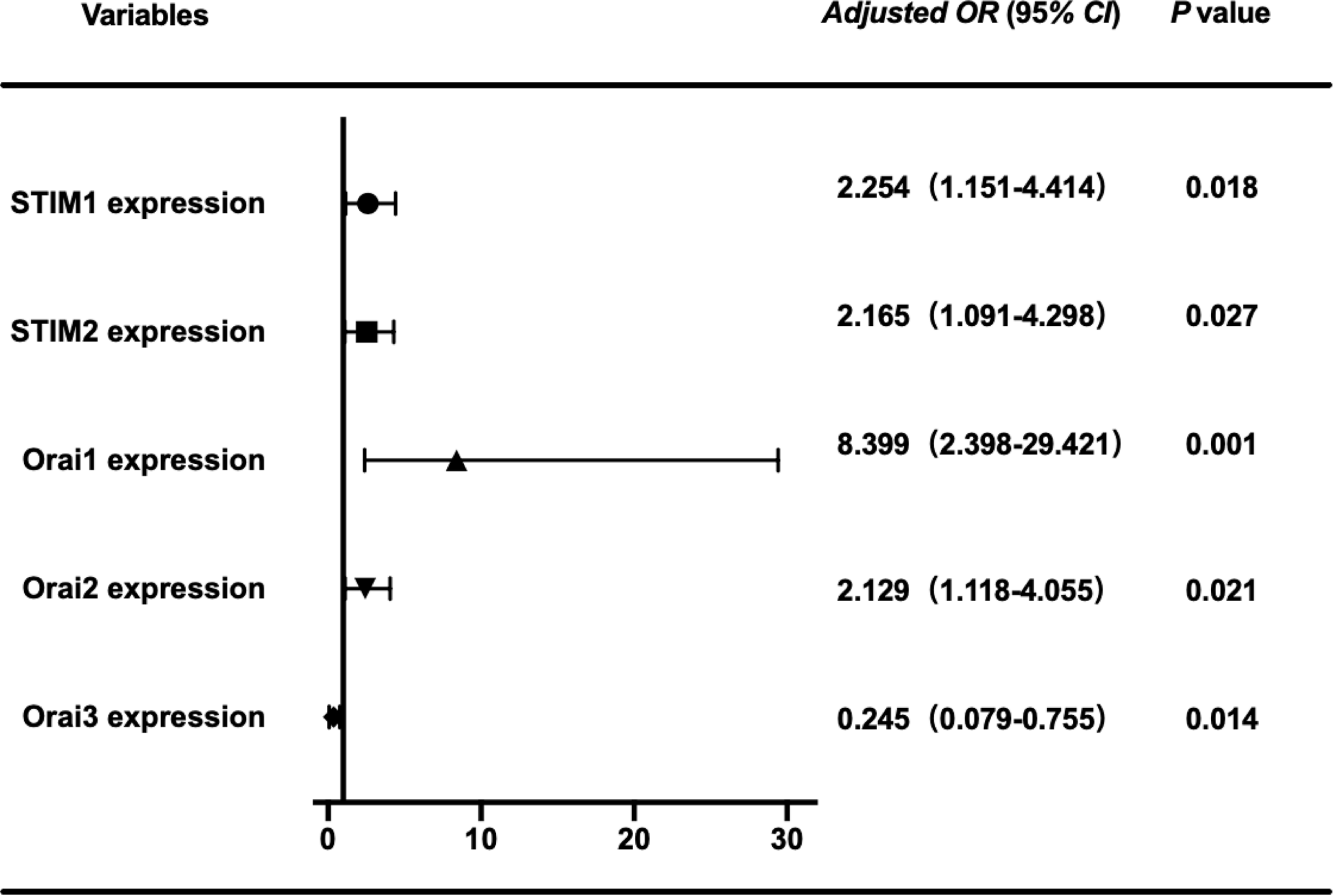
Logistic regression analysis of gene expression after adjusting for age and BMI.

To eliminate the influences of age and BMI on the expression of genes, we used binary logistic regression model for analysis. The binary logistic regression analysis results of SOCE gene mRNA expression after adjusted using age and BMI were summarized in **Table 3** and **Figure 2**. Logistic regression showed STIM1 (adjusted OR: 2.253, 95% CI: 1.151–4.414, *P* = 0.018), STIM2 (adjusted OR: 2.165, 95% CI: 1.091–4.298, *P* = 0.027), Orai1 (adjusted OR: 8.399, 95% CI: 2.398–29.421, *P* = 0.001), Orai2 (adjusted OR: 2.129, 95% CI: 1.118–4.055, *P* = 0.021), and Orai3 (adjusted OR: 0.245, 95% CI: 0.079–0.755, *P* = 0.014) were factors contributing to the incidence of PCOS.

**Table 3:** Logistic regression analysis of gene expression after adjusting for age and BMI.

Variables	*B*	*SE*	*Wald*	*P*	*Adjusted OR*	95*% CI*	
						*Lower*	*Upper*
STIM1 expression	0.813	0.343	5.616	0.018	2.254	1.151	4.414
STIM2 expression	0.772	0.35	4.875	0.027	2.165	1.091	4.298
Orai1 expression	2.128	0.64	11.07	0.001	8.399	2.398	29.421
Orai2 expression	0.756	0.329	5.286	0.021	2.129	1.118	4.055
Orai3 expression	−1.409	0.575	5.996	0.014	0.245	0.079	0.755

### Correlation Analysis Between Gene Expression Level and Baseline Data of Clinical Samples

**Figure 3:**
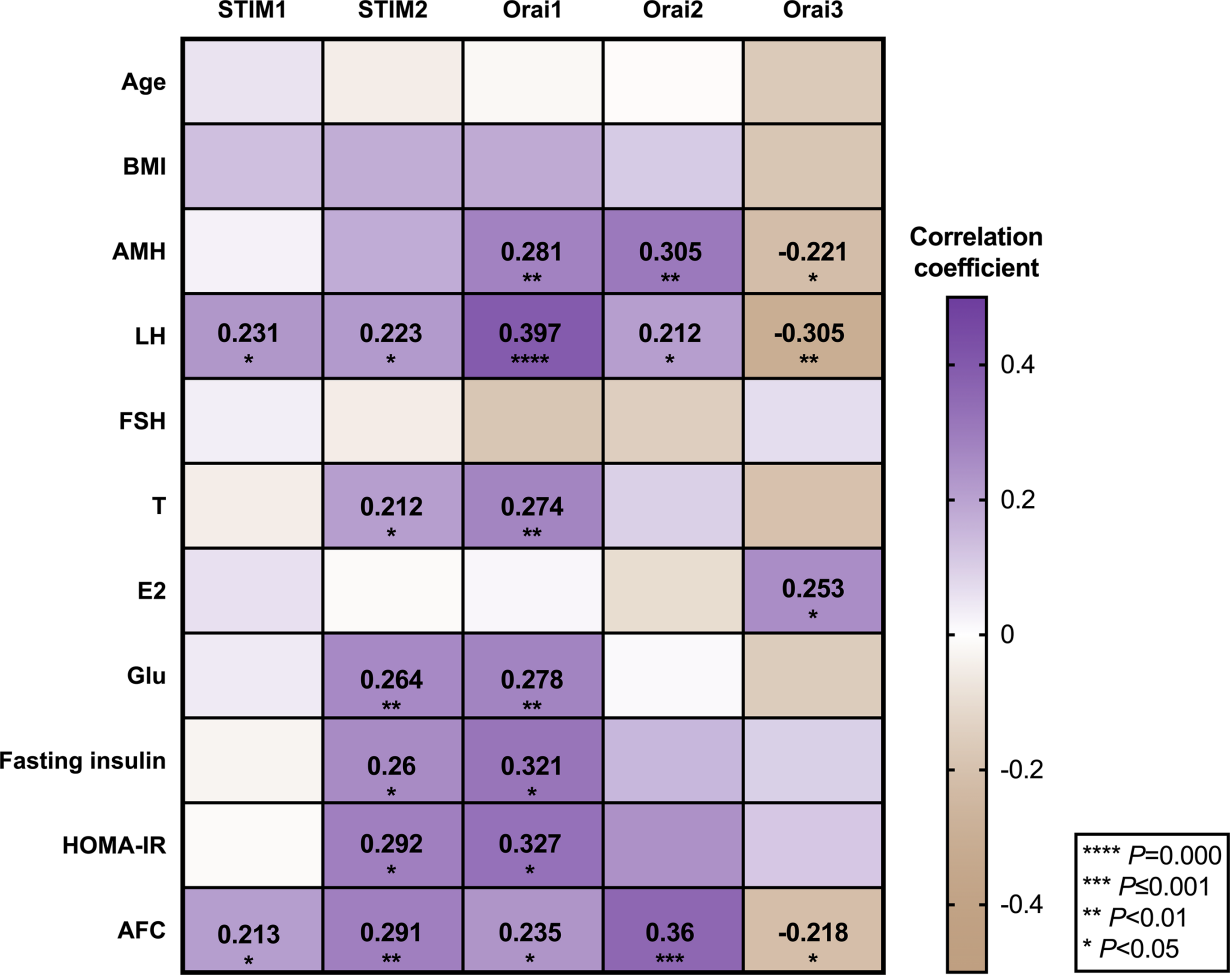
Correlation between PCOS phenotypes and relative mRNA expression of SOCE genes in GCs of participants.

To determine whether the expression level of genes is related to clinical endocrine and glucose metabolism characters of corresponding samples, we performed Pearson correlation analysis to elucidate the relationship between SOCE and various PCOS phenotypes by investigating the correlation between each differentially expressed mRNA levels of SOCE molecules and phenotype. As shown in **Figure 3**, STIMs and Orais mRNA levels showed significant correlation with LH and AFC, and STIM2 and Orai1 mRNA levels showed positive correlation with T, glucose, fasting insulin, and HOMA-IR. Futhermore, Orai1 and Orai2 mRNA levels in the GCs of patients with PCOS were positively correlated with AMH. In addition, The Orai3 exhibited a negative correlation with AMH while positively correlated with E2. In addition, we presented the exact correlation analysis data of all the measured indicators in **Supplementary Table 2**.

## Discussion

Little is known regarding the functional role of SOCE in the pathology of PCOS; here, we analyzed the expression of several core genes of SOCE in GCs of PCOS patients for the first time. Our finding indicated that the SOCE signaling was determined in PCOS GCs, where STIM1, STIM2, Orai1, and Orai2 significantly increased, whereas Orai3 decreased. Moreover, STIMs and Orais were defined as the main factors affecting PCOS after adjusted by age and BMI. The expression levels of the five genes were mainly correlated with the hormone levels and AFC.

Calcium signaling is essential to regulate cellular activities including hormone secretion, proliferation, or apoptosis (6). It is important to maintain intracellular Ca2+ homeostasis, for abnormal Ca2+ fluctuations could induce rare and common disorders (14). SOCE is a sophisticated mechanism playing an important role in rescuing intracellular calcium oscillation (12, 13). In terms of cellular Ca2+ homeostasis, the ER plays a prime role in the process that ensuring proper protein folding which usually happened under ER stress (23, 24). It seems that SOCE could establish a certain connection with ER stress in PCOS.

In our present study, we hypothesized that the possible mechanisms for linking SOCE to pathogenesis of PCOS could be its protective effect on adapting ER stress and its ability on enhancing the proliferation signal of GCs. Under ER stress, STIMs activate and couple to Orai1 to rescue Ca2+ oscillation in ER, and then SOCE channel opens. In present study, we found that STIMs, Orai1 and Orai2 were upregulated in GCs of PCOS, so it could be speculated that SOCE signaling was activated in the pathogenesis PCOS. Takahashi et al. (25) demonstrated that ER stress was activated in GCs of both patients with PCOS and the dehydroepiandrosterone (DHEA)-treated mouse model of PCOS. Unanimously, in the study of Azhary and colleagues (26), they proved that androgens activate ER stress, thereby enhancing the apoptosis of GCs and resulting in the occurrence of PCOS. However, the sample amounts in this study were too small to support that the apoptosis of GCs was the cause of PCOS. Only the prolonged ER stress is accounted for apoptosis of cells (27). Investigators showed that ER stress activated an adaptive program termed unfolded protein response to deal with the accumulated misfolded proteins and thus relieved ER dysfunction (28, 29). Thus, we assumed that in the pathology of PCOS, ER Ca2+ was consumed in the state of ER stress accompanied by oscillation of cytosolic free Ca2+, and the SOCE was activated as an adaptive program in cells with the upregulated expression of STIM1, Orai1, STIM2, and Orai2. Moreover, elevated STIM1 and Orai1 expressions were thought to promote tumor cell proliferation in various cancers (30). Studies have proved that GCs proliferation was increased in the pathogenesis of PCOS (19). Taken together, the adaptive response of SOCE to ER stress in GCs may thereby lead to increased cell proliferation. In addition, the binary logistic regression analysis demonstrated STIMs and Orais were factors affecting PCOS after adjusted by BMI and age. The precise functional mechanism of SOCE in the onset and progression of PCOS remains to be explored.

Insulin resistance and compensatory hyperinsulinemia are often regarded as the main defects in women with PCOS (31). Hyperinsulinemia may further promote abnormal androgen secretion (32, 33). Moreover, it is demonstrated that SOCE has could be triggered at the stage of ER stress to increase the secretion of insulin even under sub-threshold glucose (34). Interestingly, insulin could conversely upregulate Orais (35). These studies linked insulin with SOCE. Thus, we analyzed the correlation between the expression levels of SOCE genes and glucose metabolism level as well as testosterone, and we found that expression level of Orai1 showed significant correlation with GLU, fasting insulin, and HOMA-IR. Moreover, STIM2 and Orai1 showed significant correlation with testosterone, respectively. However, the precise mechanisms still need to be fully described. SOCE through ORAI channels triggered by STIM1 is a major mechanism, mediating the signals of many hormones, growth factors, and neurotransmitters (36, 37). Tal et al. (38) found that the concentration of placental growth factor (PlGF) protein in follicular fluid from PCOS women was higher compared with controls and showed a positive correlation with AMH. Researchers also observed that PlGF could promote proliferation in ovary carcinoma cells by upregulating SOCE signal (39). Interestingly, hypothalamic luteinizing hormone-releasing hormone (LHRH) controls reproductive axis by promoting gonadotropin release, and researchers found that LHRH could activate Orai1 channels in mouse gonadotroph (40). The present study revealed that several SOCE elements showed correlation with AMH and LH, which are common characters of PCOS.

Many research studies recently indicated SOCE as a reliable therapeutic target and SOCE blocker might be of therapeutic benefit that the application of specific SOCE blockers would not affect other normal cells. Blocking SOCE could alleviate inflammation (41, 42), Zhang et al. (43) found that knocking down STIM1 in neutrophils could produce the same effect, which was due to reducing the production of reactive oxygen species (ROS). Furthermore, investigators suggested that inhibition of SOCE could block the proliferation of megakaryocytes in patients with myeloproliferative tumors (44). Clinical studies found increased glycolysis in women with PCOS (45). A new research showed that STIM1 promotes glycolysis and FAS during cell proliferation, and STIM1 was considered to be a new metabolic checkpoint (46). These results suggested that SOCE might be a possible target for the treatment and examination of PCOS. In short, there have been successful applications of SOCE blockers in clinical trials (47), but more research studies are needed to improve the treatment strategy.

The present study was the first to prove the several mRNA expressions level of SOCE elements of GCs in women with PCOS and to show significant relationship between these genes and PCOS characters. In addition, the expression levels of the STIMs and Orais were performed as major factors affecting PCOS after adjusted by age and BMI. Thus, we speculated SOCE could regulate cellular Ca2+ concentration and then took part in proliferation, and hormone secretion of GCs. Combining our findings with more detailed mechanistic and pharmacological PCOS studies may disclose additional valuable information and perhaps novel treatment strategies. However, several limitations still exist. First, GCs obtained before LH/hCG stimulation would be more appropriate in our study. However, it is very difficult to collect abundant immature GCs at gynecologic surgery. Second, the sample amount of the present study was relatively small. The results still need to be verified in a larger population.

## Conclusion

In summary, for the first time, our findings indicated that SOCE was differently expressed, where STIM1, STIM2, Orai1, and Orai2 significantly increased, whereas Orai3 decreased in PCOS GCs, which might be dominantly involved in dysfunction of ovarian GCs and hormone changes in PCOS. However, further studies are required to investigate function and regulation of SOCE in PCOS.

## Data Availability Statement

The original contributions presented in the study are included in the article/**Supplementary Material**. Further inquiries can be directed to the corresponding author.

## Ethics Statement

The study was conducted according to the guidelines of the Declaration of Helsinki, and approved by the Ethics Committee of Reproductive Medicine of Shandong University with approval number 132, on December 19, 2019.

## Author Contributions

YS conceived and designed this study. TS contributed to experiments, statistical analysis, interpretation of data, and draft of the manuscript. PL and QW performed statistical analysis and participated in the discussion. BH and YW acquired the data. YB analyzed and interpreted the data. YS participated in the discussion and critically revised the manuscript. All authors contributed to the article and approved the submitted version.

## Funding

National Key Research and Development Program of China (2021YFC2700404) and National Key R&D Program of China (2018YFC1003202).

## Conflict of Interest

The authors declare that the research was conducted in the absence of any commercial or financial relationships that could be construed as a potential conflict of interest.

## Publisher’s Note

All claims expressed in this article are solely those of the authors and do not necessarily represent those of their affiliated organizations, or those of the publisher, the editors and the reviewers. Any product that may be evaluated in this article, or claim that may be made by its manufacturer, is not guaranteed or endorsed by the publisher.
