# Corrigendum: Comprehensive assessment of the STIMs and Orais expression in polycystic ovary syndrome

**DOI:** 10.3389/fendo.2022.1005894

**Published:** 2022-08-16

**Authors:** Tian Song, Ping Li, Qiumin Wang, Baozhen Hao, Ying Wang, Yuehong Bian, Yuhua Shi

**Affiliations:** ^1^ Center for Reproductive Medicine, Cheeloo College of Medicine, Shandong University, Jinan, China; ^2^ Key laboratory of Reproductive Endocrinology of Ministry of Education, Shandong University, Jinan, China; ^3^ Shandong Key Laboratory of Reproductive Medicine, Jinan, China; ^4^ Shandong Provincial Clinical Research Center for Reproductive Health, Jinan, China; ^5^ National Research Center for Assisted Reproductive Technology and Reproductive Genetics, Shandong University, Jinan, China; ^6^ Department of Reproductive Medicine, Women and Children’s Hospital, School of Medicine, Xiamen University, Xiamen, China; ^7^ Xiamen Key Laboratory of Reproduction and Genetics, Xiamen, China; ^8^ Shandong Provincial Maternal and Child Health Care Hospital, Cheeloo College of Medicine, Shandong University, Jinan, China; ^9^ Guangdong Provincial People’s Hospital, Guangzhou, China

**Keywords:** polycystic ovary syndrome, store-operated Ca2+ entry, STIM1, Orai1, STIM2

In the published article, an author name was incorrectly written as Qiuming Wang. The correct spelling is Qiumin Wang.

The authors apologize for this error and state that this does not change the scientific conclusions of the article in any way. The original article has been updated.

## Publisher’s note

All claims expressed in this article are solely those of the authors and do not necessarily represent those of their affiliated organizations, or those of the publisher, the editors and the reviewers. Any product that may be evaluated in this article, or claim that may be made by its manufacturer, is not guaranteed or endorsed by the publisher.

