# Supplementary material for: Comprehensive Assessment of the STIMs and Orais Expression in Polycystic Ovary Syndrome
Source: Front Endocrinol (Lausanne). 2022 May 20;13:874987. doi: 10.3389/fendo.2022.874987 (PMC9165061; doi:10.3389/fendo.2022.874987)
Supplement: Supplementary file 1 [file Table_1.docx]

**Supplementary table 1a. Clinical and endocrine parameters of PCOS patients and controls in each gene group**

|  | STIM1 | |  | STIM2 | |  |
| --- | --- | --- | --- | --- | --- | --- |
| Basic parameters | PCOS  (*n*=50) | Control  (*n*=50) | P value | PCOS  (*n*=50) | Control  (*n*=50) | P value |
| Age (years) | 30.1±3.98 | 30.14±4.54 | NS | 30.33±3.87 | 30.59±4.65 | NS |
| BMI (kg/m^2^) | 24.66±2.97 | 22.09±3.46 | 0 | 25.24±3.42 | 22.39±2.92 | 0 |
| AMH (ng/mL) | 8.24±4.26 | 3.79±1.66 | 0 | 7.99±4.21 | 4.16±1.83 | 0 |
| LH (IU/L) | 9.5±4.72 | 5.89±4.76 | 0 | 8.34±3.63 | 6.59±8.13 | 0.001 |
| FSH (U/L) | 5.73±1.84 | 6.64±1.62 | 0.004 | 5.48±1.36 | 6.52±1.64 | 0.001 |
| T (ng/dL) | 42.59±20.42 | 24.09±10.51 | 0 | 36.01±21.05 | 26.08±11.79 | 0.001 |
| E2 (pg/mL) | 52.1±71.26 | 38±16.33 | NS | 39.78±19.05 | 39.73±15.99 | NS |
| GLU (mmol/L) | 5.36±0.46 | 5.2±0.41 | NS | 5.35±0.41 | 5.21±0.38 | NS |
| Fasting Insulin (mIU/L) | 21.95±26.85 | 15.6±15.88 | 0.03 | 22.13±27.01 | 15.43±10.74 | NS |
| HOMA-IR | 4.89±4.14 | 3.66±3.92 | 0.02 | 4.88±4.15 | 3.53±2.35 | NS |
| AFC | 27.02±12.22 | 16.78±5.77 | 0 | 28.63±13.23 | 15.82±5.43 | 0 |

**Supplementary table 1b. Clinical and endocrine parameters of PCOS patients and controls in each gene group**

|  | Orai1 | |  | Orai2 | |  | Orai3 | |  |
| --- | --- | --- | --- | --- | --- | --- | --- | --- | --- |
| Basic parameters | PCOS  (*n*=50) | Control  (*n*=50) | *P* value | PCOS  (*n*=50) | Control  (*n*=50) | *P* value | PCOS  (*n*=50) | Control  (*n*=50) | *P* value |
| Age (years) | 30.35±3.69 | 30.38±4.65 | NS | 30.54±3.96 | 30.67±4.46 | 0.878 | 30.29±3.83 | 30.73±4.47 | NS |
| BMI (kg/m^2^) | 24.6±3.49 | 22.79±3.86 | 0.019 | 24.59±3.01 | 22.53±3.02 | 0.001 | 25.19±3.17 | 22.67±2.88 | 0 |
| AMH (ng/mL) | 7.83±3.97 | 3.99±1.65 | 0 | 8.26±4.19 | 4.15±1.84 | 0 | 8.06±4.11 | 4.06±1.81 | 0 |
| LH (IU/L) | 9.32±4.64 | 5.82±4.78 | 0 | 8.22±3.71 | 6.69±8.13 | 0.003 | 8.45±3.74 | 6.54±8.12 | 0 |
| FSH (U/L) | 5.91±1.74 | 6.54±1.54 | 0.031 | 5.43±1.34 | 6.42±1.7 | 0.002 | 5.56±1.35 | 6.35±1.71 | 0.013 |
| T (ng/dL) | 39.69±19.19 | 23.89±10.48 | 0 | 36.09±20.91 | 26.47±11.64 | 0.002 | 36.52±21.11 | 26.25±11.87 | 0.001 |
| E2 (pg/mL) | 52.73±72.48 | 39.6±17.35 | NS | 40.54±19.79 | 40.3±16.59 | NS | 39.22±19.27 | 42.26±19.15 | NS |
| GLU (mmol/L) | 5.33±0.45 | 5.26±0.4 | NS | 16.33±76.46 | 5.21±0.38 | NS | 5.34±0.4 | 5.18±0.37 | 0.042 |
| Fasting Insulin (mIU/L) | 22.81±28.44 | 20.88±17.89 | NS | 21.59±27.6 | 13.34±6.76 | 0.034 | 22.17±26.61 | 14.12±10.22 | 0.011 |
| HOMA-IR | 5.37±6.16 | 4.85±4.26 | NS | 4.92±4.32 | 3.09±1.62 | 0.014 | 4.9±4.08 | 3.23±2.29 | 0.005 |
| AFC | 23.48±6.98 | 16.61±5.98 | 0 | 27.73±12.45 | 15.64±5.47 | 0 | 28.65±13.27 | 15.5±5.16 | 0 |

NS means not statistically significant
