# Supplementary material for: Comprehensive Assessment of the STIMs and Orais Expression in Polycystic Ovary Syndrome
Source: Front Endocrinol (Lausanne). 2022 May 20;13:874987. doi: 10.3389/fendo.2022.874987 (PMC9165061; doi:10.3389/fendo.2022.874987)
Supplement: Supplementary file 2 [file Table_2.docx]

Supplementary Table 2 Correlation of gene expression levels and phenotype of clinical samples

| Clinical phenotype | Genes | Pearson r | P value |
| --- | --- | --- | --- |
| Age | STIM1  STIM2  Orai1  Orai2  Orai3 | \| 0.056 \| \| --- \| \| -0.049 \| \| -0.022 \| \| -0.011  -0.167 \| | \| 0.579 \| \| --- \| \| 0.636 \| \| 0.829 \| \| 0.913  0.102 \| |
| BMI | STIM1  STIM2  Orai1  Orai2  Orai3 | \| 0.132 \| \| --- \| \| 0.176 \| \| 0.181 \| \| 0.109  -0.181 \| | \| 0.201 \| \| --- \| \| 0.093 \| \| 0.082 \| \| 0.302  0.084 \| |
| AMH | STIM1  STIM2  Orai1**^#^**  Orai2**^#^**  Orai3**^#^** | \| 0.027 \| \| --- \| \| 0.179 \| \| 0.281 \| \| 0.305  -0.221 \| | \| 0.794 \| \| --- \| \| 0.086 \| \| 0.005 \| \| 0.003 \|   0.033 |
| LH | STIM1**^#^**  STIM2**^#^**  Orai1**^#^**  Orai2**^#^**  Orai3**^#^** | \| 0.231 \| \| --- \| \| 0.223 \| \| 0.397 \| \| 0.212 \|   -0.305 | \| 0.025 \| \| --- \| \| 0.032 \| \| <0.0001 \| \| 0.041 \|   0.003 |
| FSH | STIM1  STIM2  Orai1  Orai2  Orai3 | \| 0.032 \| \| --- \| \| -0.05 \| \| -0.183 \| \| -0.15  0.069 \| | \| 0.756 \| \| --- \| \| 0.623 \| \| 0.075 \| \| 0.143  0.501 \| |
| T | STIM1  STIM2**^#^**  Orai1**^#^**  Orai2  Orai3 | \| -0.048 \| \| --- \| \| 0.212 \| \| 0.274 \| \| 0.099  -0.203 \| | \| 0.637 \| \| --- \| \| 0.037 \| \| 0.007 \| \| 0.344  0.051 \| |
| E2 | STIM1  STIM2  Orai1  Orai2  Orai3**^#^** | \| 0.062 \| \| --- \| \| -0.015 \| \| 0.017 \| \| -0.104 \|   0.253 | \| 0.54 \| \| --- \| \| 0.886 \| \| 0.866 \| \| 0.313  0.015 \| |
| Glu | STIM1  STIM2**^#^**  Orai1**^#^**  Orai2  Orai3 | \| 0.042 \| \| --- \| \| 0.264 \| \| 0.278 \| \| 0.012  -0.160 \| | \| 0.679 \| \| --- \| \| 0.009 \| \| 0.006 \| \| 0.911  0.120 \| |
| Fasting insulin | STIM1  STIM2**^#^**  Orai1**^#^**  Orai2  Orai3 | \| -0.031 \| \| --- \| \| 0.26 \| \| 0.321 \| \| 0.152  0.100 \| | \| 0.821 \| \| --- \| \| 0.041 \| \| 0.021 \| \| 0.239  0.418 \| |
| HOMR-IR | STIM1  STIM2**^#^**  Orai1**^#^**  Orai2  Orai3 | \| -0.015 \| \| --- \| \| 0.292 \| \| 0.327 \| \| 0.244  0.112 \| | \| 0.908 \| \| --- \| \| 0.021 \| \| 0.018 \| \| 0.063  0.365 \| |
| AFC | STIM1**^#^**  STIM2**^#^**  Orai1**^#^**  Orai2**^#^**  Orai3**^#^** | \| 0.213 \| \| --- \| \| 0.291 \| \| 0.235 \| \| 0.36  -0.218 \| | \| 0.04 \| \| --- \| \| 0.004 \| \| 0.023 \| \| <0.001  0.035 \| |

**^#^** Statistical significance
